# Near-IR Luminescence of Rare-Earth Ions (Er^3+^, Pr^3+^, Ho^3+^, Tm^3+^) in Titanate–Germanate Glasses under Excitation of Yb^3+^

**DOI:** 10.3390/ma15103660

**Published:** 2022-05-20

**Authors:** Karolina Kowalska, Marta Kuwik, Joanna Pisarska, Wojciech A. Pisarski

**Affiliations:** Institute of Chemistry, University of Silesia, Szkolna 9 Street, 40-007 Katowice, Poland; marta.kuwik@us.edu.pl (M.K.); joanna.pisarska@us.edu.pl (J.P.)

**Keywords:** germanate glasses, ytterbium ions, energy transfer, luminescence property relationship

## Abstract

Inorganic glasses co-doped with rare-earth ions have a key potential application value in the field of optical communications. In this paper, we have fabricated and then characterized multicomponent TiO_2_-modified germanate glasses co-doped with Yb^3+^/Ln^3+^ (Ln = Pr, Er, Tm, Ho) with excellent spectroscopic properties. Glass systems were directly excited at 980 nm (the ^2^F_7/2_ → ^2^F_5/2_ transition of Yb^3+^). We demonstrated that the introduction of TiO_2_ is a promising option to significantly enhance the main near-infrared luminescence bands located at the optical telecommunication window at 1.3 μm (Pr^3+^: ^1^G_4_ → ^3^H_5_), 1.5 μm (Er^3+^: ^4^I_13/2_ → ^4^I_15/2_), 1.8 μm (Tm^3+^: ^3^F_4_ → ^3^H_6_) and 2.0 μm (Ho^3+^: ^5^I_7_ → ^7^I_8_). Based on the lifetime values, the energy transfer efficiencies (η_ET_) were estimated. The values of η_ET_ are changed from 31% for Yb^3+^/Ho^3+^ glass to nearly 53% for Yb^3+^/Pr^3+^ glass. The investigations show that obtained titanate–germanate glass is an interesting type of special glasses integrating luminescence properties and spectroscopic parameters, which may be a promising candidate for application in laser sources emitting radiation and broadband tunable amplifiers operating in the near-infrared range.

## 1. Introduction

Numerous inorganic glass materials are fabricated and widely applied industrially. Novel glass host matrices are still developed and must fulfill the rising demand for good-quality optical components and devices such as active optical fibers, solid-state laser sources, broadband near-IR fiber amplifiers, photonic integrated devices, and so on [1,2,3,4]. Systematic studies clearly demonstrate that positions and spectral linewidths for characteristic luminescence bands of lanthanides ions can be tuned according to the chemical compositions of glass and glass-ceramic matrices and dopant ions [5,6,7,8,9]. One of the most perspective options to remedy the drawbacks and improve the photoluminescence properties of lanthanide ions is a modification of the glassy network with suitable additives. In this context, TiO_2_-modified glasses [10,11] have gained much importance due to their interesting properties that make them promising candidates for luminescent sources and optical devices. The beneficial effect of these additives is the improvement of the thermal and chemical stability of glasses [12,13,14]. It is also assumed that presence of titanium dioxide in matrices with low phonon energy may significantly broaden and enhance the luminescence bands of lanthanides ions.

From the accumulated experience and literature data [15,16], it is known that the trivalent ytterbium ions have been extensively studied for use as efficient emitters of radiation in the infrared range. It should be noted that Yb^3+^ ions with a broad absorption band in the wavelength region of 860–1060 nm and a relatively long fluorescence lifetime (1–2 ms) can be excellent sensitizers to activate other lanthanide ions for luminescence [17,18]. For this reason, co-doping of materials with Yb^3+^ ions allows for the efficient pumping of around 980 nm using a commercially available diode [19,20]. From the experimental approach, it can be concluded the near-IR radiative transitions of lanthanide ions are greatly dependent on the reduction of matrix phonons to achieve high luminescence efficiency. Indeed, in the past few years, subsequent research on germanate glass remains a perspective option as an oxide glass host matrix for lanthanide ions thanks to its favorable properties, such as smaller multiphonon relaxation probabilities due to relatively low phonon energy (~800 cm^−1^), high transparency from the visible to the infrared region and presence of non-linear optical effects [21,22]. One should remember that the following lanthanides Ln^3+^ ions such as Pr^3+^, Er^3+^, Tm^3+^, and Ho^3+^ are proposed as co-activators for photoluminescence in Yb^3+^/Ln^3+^-doubly doped glasses owing to their favorable location of energy levels and the possibility of radiative transitions at the infrared [23]. Among them, Tm^3+^ [24] has gained increasing attention because the near-IR emission associated with the ^3^F_4_ → ^3^H_6_ transition at 1800 nm is useful as a medical light source. The Er^3+^ ions [25] are widely used in materials for optical applications. Main ^4^I_13/2_ → ^4^I_15/2_ near-IR transition of Er^3+^ at 1500 nm corresponds to the C + L telecommunication window. Next, Ho^3+^ ions [26] are one of the interesting dopants appropriate for laser sources operated at 2000 nm owing to the ^5^I_7_ → ^7^I_8_ transition. Although such interesting observations, insufficient attention has been paid to the effects of TiO_2_ on near-IR emission properties of low-phonon germanate glasses co-doped with lanthanides. From this point of view, it is interesting to find out how emission bands of selected lanthanide ions located in the near-IR range are changed with different GeO_2_:TiO_2_ molar ratios in chemical composition under Yb^3+^ ions excitation.

This paper concerns novel multicomponent titanate–germanate glasses belonging to the low-phonon oxide glass family. Glass samples were successfully synthesized using the conventional high-temperature melting technique. The optical properties of glass series containing two network-formers, TiO_2_ and GeO_2_, were characterized using luminescence spectroscopy. In our studies, Yb^3+^ plays an important role, such as a sensitizer to activate selected lanthanide ions. Our attention has been paid to titanate–germanate glass systems co-doped with Yb^3+^/Er^3+^, Yb^3+^/Ho^3+^, Yb^3+^/Tm^3+^, Yb^3+^/Pr^3+^, and their energy transfer process. Near-infrared luminescence spectra and decay curves were examined for samples where GeO_2_ was substituted by TiO_2_. In the studied glass systems, molar ratios are changed from GeO_2_:TiO_2_ = 5:1 up to GeO_2_:TiO_2_ = 1:5. Based on the measured values of luminescence lifetimes, the energy transfer efficiencies Yb^3+^ → Ln^3+^ (Ln = Pr Er, Tm, and Ho) were determined for glass samples differing in TiO_2_ content. The influence of TiO_2_ on structural properties has already been carried out on the previous titanate–germanate glasses published recently [27].

## 2. Materials and Methods

The investigated TiO_2_-modified germanate glasses co-doped with lanthanides ions with chemical composition (given in mol%): xTiO_2_-(60−x)GeO_2_-30BaO-9.5Ga_2_O_3_-0.5Yb_2_O_3_ and xTiO_2_-(60−x)GeO_2_-30BaO-9.4Ga_2_O_3_-0.5Yb_2_O_3_-0.1Ln_2_O_3_, (where Ln = Er, Tm, Pr, Ho, and x = 10, 20, 30, 40, 45, 50) were obtained by traditional melt quenching technique. In present research, glasses containing various molar ratios GeO_2_:TiO_2_ are equal to 5:1, 2:1, 1:1, 1:2, 1:3, and 1:5 and glass codes are as follows: 5Ge-1Ti, 2Ge-1Ti, 1Ge-1Ti, 1Ge-2Ti, 1Ge-3Ti, and 1Ge-5Ti. All of the glass components used during synthesis were of high purity (99.99%) from Aldrich Chemical Co. (St. Louis, MO, USA). Appropriate precursor metal oxides were mixed in an agate mortar. After homogenization of the components, 5 g glass bathes were placed in a platinum crucible (Łukasiewicz Research Network, Institute of Ceramics and Building Materials, Cracow, Poland). In the present procedure, the melting conditions were T = 1250 °C for 60 min in an electric furnace. Finally, each glass sample was cooled to room temperature and polished to meet the requirements for optical measurements. A series of transparent glass samples with the dimensions 12 mm × 12 mm and thickness ±3 mm was successfully prepared to determine their optical properties. The luminescence measurements of glasses were performed on a Photon Technology International (PTI) Quanta-Master 40 (QM40) UV/VIS Steady State Spectrofluorometer (Photon Technology International, Birmingham, NJ, USA) supplied with a tunable pulsed optical parametric oscillator (OPO) pumped by the third harmonic of an Nd:YAG laser (Opotek Opolette 355 LD, OPOTEK, Carlsbad, CA, USA). The laser system was coupled with a 75 W xenon lamp, a double 200 mm monochromator, and a Hamamatsu H10330B-75 detector (Hamamatsu, Bridgewater, NJ, USA). The emission spectra were recorded with a spectral resolution of 0.5 nm. Decay curves were recorded by a PTI ASOC-10 (USB-2500) oscilloscope with an accuracy of ±2 μm and have been measured under excitation wavelengths 980 nm and monitoring emission wavelength 1030 nm. In order to compare the emission intensity under the same experimental conditions, measurements of glass systems were carried out at the same slit settings. Measurements were performed at room temperature.

## 3. Results and Discussion

### 3.1. Optical Absorption Properties

**Figure 1:**
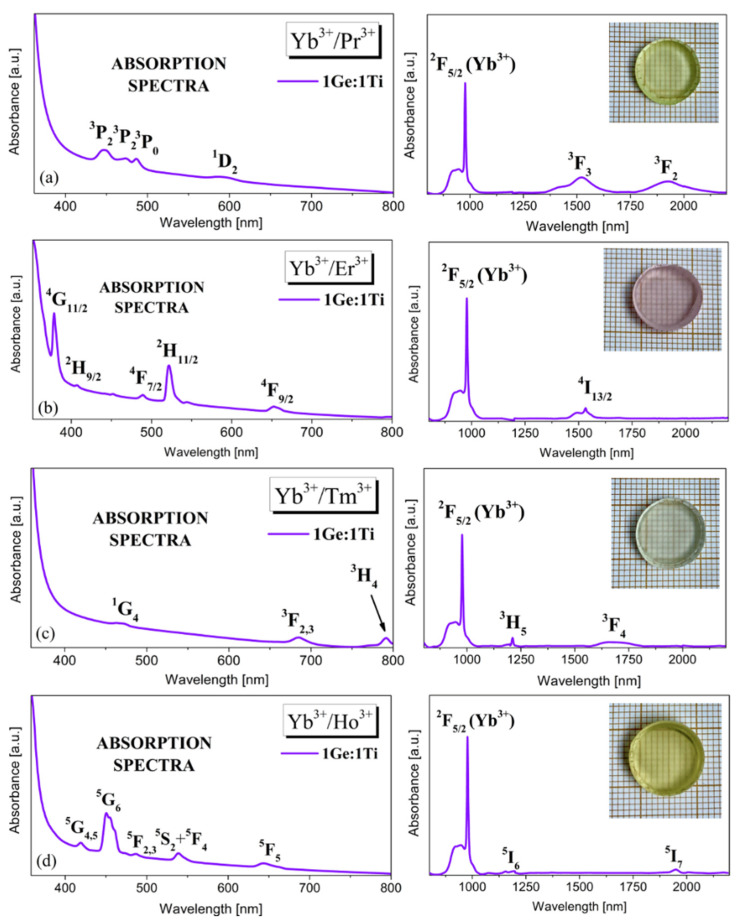
Typical absorption spectra of titanate–germanate glasses co-doped with Yb^3+^/Pr^3+^ (**a**), Yb^3+^/Er^3+^ (**b**), Yb^3+^/Tm^3+^ (**c**) and Yb^3+^/Ho^3+^ (**d**). Inset shows a photographic image of the glass sample.

In this study, measurements of the absorption spectra of glass systems 1Ge:1Ti co-doped with Yb^3+^/Ln^3+^ (Ln = Er, Pr, Tm, Ho) were carried out and presented in Figure 1.

Absorption spectra measured for representative titanate–germanate glasses consist of the characteristic bands corresponding to transitions originating from the ground state to higher-lying excited states of selected lanthanide ions. The glass sample co-doped with Yb^3+^/Pr^3+^ ions (Figure 1a) shows four weakly intense absorption bands in the 350–800 nm range. The two most intense absorption bands due to ^3^H_4_ → ^3^F_3_ and ^3^H_4_ → ^3^F_2_ transitions are well observed in the infrared spectral range. Interestingly, literature data indicate that on the band edge, due to ^3^H_4_ → ^3^F_3_, a weakly separated absorption band at about 1400 nm associated with the ^3^H_4_ → ^3^F_4_ transition is detected [28]. Next, it is evidently seen for titanium germanium glass co-doped with Yb^3+^/Er^3+^ that the absorption bands (Figure 1b) due to the transition from the ^4^I_15/2_ state of Er^3+^ are well observed at 380 nm (^4^G_11/2_), 407 nm (^2^H_9/2_), 489 nm (^4^F_7/2_), 522 nm (^2^H_11/2_), 652 nm (^4^F_9/2_) and band centered in the near-infrared range at 1530 nm due to ^4^I_15/2_ → ^4^I_13/2_ transition [29]. Figure 1c shows the absorption spectrum of the Yb^3+^/Tm^3+^ co-doped titanate–germanate sample. The five absorption bands at 471 nm, 685 nm, 790 nm, 1210 nm, and 1690 nm correspond to the transitions from the ground state ^3^H_6_ to excited stated ^1^G_4_, ^3^F_2_ + ^3^F_3_, ^3^H_4_, ^3^H_5_, and ^3^F_4_, respectively [30]. In turn, the absorption spectrum measured for Yb^3+^/Ho^3+^ co-doped glass is shown in Figure 1d. The results show that the obtained glass characterizes seven absorption bands in the spectral region of 350–2200 nm. The spectrum exhibits the bands due to the following absorption transitions: ^5^I_8_ → ^5^G_4,5_, ^5^I_8_ → ^5^G_6_, ^5^I_8_ → ^5^F_2,3_, ^5^I_8_ → ^2^S_2_+^5^F_4_, ^5^I_8_ → ^5^F_5_ in the visible range and ^5^I_8_ → ^5^I_6_, ^5^I_8_ → ^5^I_7_ in the NIR region [31]. For all glass samples, note that the main peak in the absorption spectra was concentrated at 980 nm, defining the lowest Yb^3+^: ^2^F_5/2_ Stark splitting energy level is the most intense; therefore, in the following section, the excitation line at 980 nm had been selected to investigate the near-infrared luminescence properties of the fabricated glasses co-doped with Yb^3+^/Pr^3+^, Yb^3+^/Er^3+^, Yb^3+^/Tm^3+^, Yb^3+^/Ho^3+^, where Yb^3+^ plays an important role of emission sensitizer for lanthanides ions.

### 3.2. Near-Infrared Luminescence Properties

**Figure 2:**
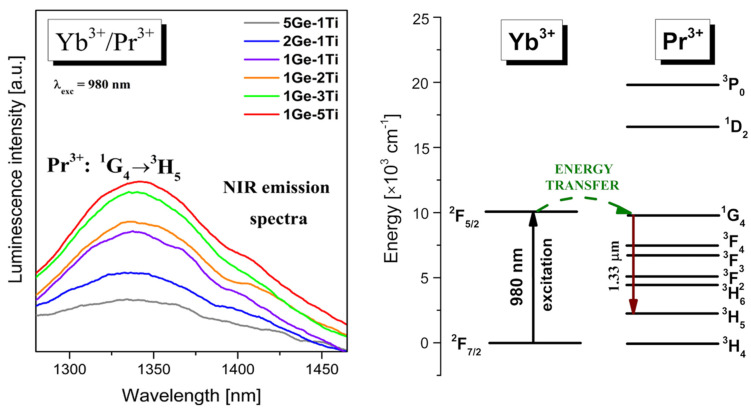
Near-infrared emission spectra and energy level diagram for titanate–germanate glasses co-doped with Yb^3+^/Pr^3+^.

Trivalent ytterbium (Yb^3+^) has a simple energy-level structure, i.e., the ^2^F_7/2_ ground level and the ^2^F_5/2_ excited level, with an energy separation between them of about 10,000 cm^−1^ [32]. The Yb^3+^ ion has been demonstrated to be an excellent emission sensitizer for other lanthanides due to its effective absorption cross-section at 980 nm [33]. In the presented work, four titanate–germanate glass systems co-doped with Yb^3+^/Pr^3+^, Yb^3+^/Er^3+^, Yb^3+^/Tm^3+^, and Yb^3+^/Ho^3+^ varying with TiO_2_ referred to as 5Ge-1Ti, 2Ge-1Ti, 1Ge-1Ti, 1Ge-2Ti, 1Ge-3Ti, and 1Ge-5Ti were selected and their near-IR emission properties under direct excitation of Yb^3+^ at wavelength 980 nm were compared. In addition, the interactions between Yb^3+^ and the second lanthanide ion and their mechanisms are discussed and presented schematically for each glass system on diagrams of the energy levels in order to understand the energy transfer processes. One of the interesting aspects of the ongoing research focusing on the properties of co-doped glasses is the observation of the sensitization of near-IR emission of Pr^3+^ at 1.35 μm under excitation Yb^3+^. Figure 2 presents near-IR luminescence spectra for Yb^3+^/Pr^3+^ co-doped titanate–germanate glasses varying with TiO_2_.

The observed near-IR emission bands at about 1.35 μm correspond to ^1^G_4_ → ^3^H_5_ transition of Pr^3+^. It should be particularly pointed out that the emission intensities of Pr^3+^ ions increased significantly with increasing TiO_2_ concentration from 5Ge-1Ti to 1Ge-5Ti. Hence, it could be suggested that the introduction of titanium dioxide to germanate glass favor near-infrared luminescence attributed to the ^1^G_4_ → ^3^H_5_ transition of Pr^3+^ under direct excitation of Yb^3+^. The observed ^1^G_4_ → ^3^H_5_ transition of Pr^3+^ ions in titanate–germanate glass under excitation of Yb^3+^ is presented on the energy level diagram in Figure 2. It is clearly seen that both excited levels ^2^F_5/2_ (Yb^3+^) and ^1^G_4_ (Pr^3+^) lie close to each other and the energy gap between them is very small. Moreover, the absorption cross-section at around 980 nm is larger for Yb^3+^ than Pr^3+^ ions, which is crucial for the pumping efficiency of praseodymium-doped fiber amplifiers PDFA [34,35]. Due to this fact, the energy transfer process Yb^3+^ → Pr^3+^ is nearly resonant and supposed to be much more efficient in titanate–germanate glass. Thus, we observe near-IR emission at 1.35 μm due to ^1^G_4_ → ^3^H_5_ transition of Pr^3+^, which enhanced rapidly with increasing in TiO_2_ content. Based on the above experiment analysis, it can be concluded that Yb^3+^/Pr^3+^ co-doped germanate glass in the presence of TiO_2_ is promising for near-IR emission and sample 1Ge-5Ti seems to be a potential precursor active glass material to realize a fiber laser operating at 1.35 μm [36,37].

**Figure 3:**
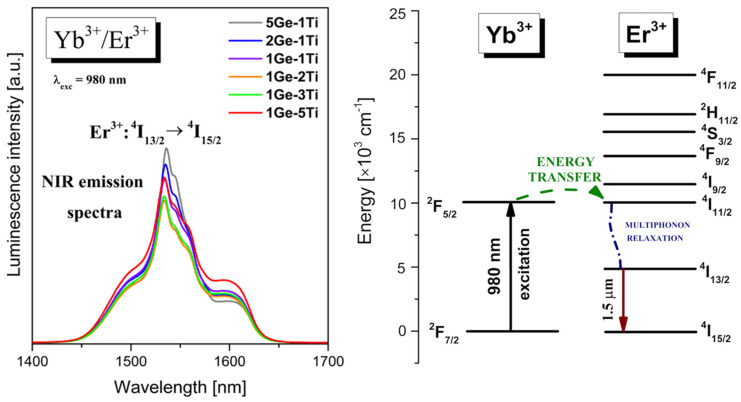
Near-infrared emission spectra and energy level diagram for titanate–germanate glasses co-doped with Yb^3+^/Er^3+^.

From the literature [38,39,40], it is well known that broadband near-infrared emission bands of Er^3+^ ions in inorganic glasses depend strongly on their chemical compositions. The rapid development of optical telecommunications requires the broadening of the near-IR range for erbium-doped fiber amplifiers (EDFA), in which signal transmission occurs at about 1500 nm. In fact, the EDFA systems based on silicate glasses [41,42] exhibit relatively narrow bandwidth, which contributes to the limited near-infrared broadband transmission. For that reason, many precursor glass systems singly doped with Er^3+^ and co-doped with Yb^3+^/Er^3+^ are still tested and selected in order to obtain enhanced near-IR luminescence in the so-called third telecommunication window. Figure 3 presents near-IR emission spectra measured in the wavelength range from 1400 nm to 1700 nm for Yb^3+^/Er^3+^ co-doped titanate–germanate glasses under excitation by a 980 nm line. Near-IR emission bands centered at about 1.53 μm correspond to the ^4^I_13/2_ → ^4^I_15/2_ transition of Er^3+^.

The intensity of the near-IR emission band at 1.5 μm is reduced from 5Ge-1Ti to 1Ge-2Ti and then increased with further increasing TiO_2_ concentration up to glass sample 1Ge-5Ti. The emission linewidth for the ^4^I_13/2_ → ^4^I_15/2_ transition of Er^3+^ ions, referred to as the full width at half maximum, is larger for sample 1Ge-5Ti (FWHM = 55 nm) than 5Ge-1Ti (FWHM = 38 nm). Schematic representation of energy levels, possible energy transfer between Yb^3+^ and Er^3+^ ions and the main near-IR laser transition of Er^3+^ ions at 1.5 μm are presented in Figure 3. Similar to the Yb^3+^/Pr^3+^ system, the excitation energy transfers resonantly very fast from the ^2^F_5/2_ (Yb^3+^) to the ^4^I_11/2_ (Er^3+^) due to a small energy mismatch between the interacting excited levels [43]. The absorption cross-section of Yb^3+^ ions at around 980 nm is higher by a factor of ten roughly than that of Er^3+^ [44], favoring an efficient Yb^3+^ → Er^3+^ energy transfer process. Next, the excitation energy relaxes nonradiatively to the ^4^I_13/2_ state by multiphonon process and consequently, we observe ^4^I_13/2_ → ^4^I_15/2_ near-IR transition of Er^3+^.

Our previous studies [27] indicate that the introduction of TiO_2_ to germanate glass resulted in higher asymmetry and better covalent bonds between rare earth and oxygens. In addition, these structural changes led to the site-to-site variation of the crystal field strength in the local environment of rare earths. It resulted in the inhomogeneous broadening of spectral lines corresponding to the presence of various sites for the optically active ions. As a consequence, the profiles of emission spectra and their values of FWHM are dependent on the symmetry, the ligand field strength, and the site-to-site variation of the Ln^3+^ local environment. This is the main reason that the spectral profiles of Er^3+^, i.e., the mission peak position, emission linewidth (FWHM), and the relative intensities of shoulders existing at about 1600 nm, are changed during the modification of glass matrices. In some cases, the glass modifiers have a minor influence on absorption properties but a strong impact on the emission cross-sections attributed to the ^4^I_13/2_ → ^4^I_15/2_ near-infrared transition of Er^3+^ ions. Recently, it was well demonstrated for Er^3+^ ions in silicate glass with various Al_2_O_3_ content [45] and Er^3+^/Yb^3+^ co-doped phosphate glass modified by Y_2_O_3_ [46].

**Figure 4:**
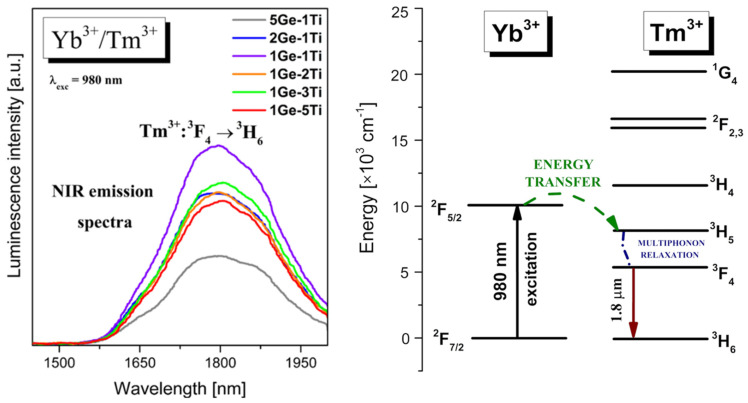
Near-infrared emission spectra and energy level diagram for titanate–germanate glasses co-doped with Yb^3+^/Tm^3+^.

Thulium is another well-known lanthanide dopant, which is introduced to various glass systems in order to generate near-IR luminescence at about 1.8 μm [47]. In particular, co-doping with Tm^3+^ and Yb^3+^ ions is an excellent way to achieve enhanced near-infrared emission by using a 980 nm wavelength as an excitation source [48]. Figure 4 shows near-IR emission spectra of series titanate–germanate glass samples co-doped with Yb^3+^/Tm^3+^. Near-IR emission bands centered at 1.8 μm are associated with ^3^F_4_ → ^3^H_6_ transition of Tm^3+^. The intensities of near-IR emission bands increase and then decrease with increasing TiO_2_ concentration in the glass composition. The highest intensity of emission band related to the ^3^F_4_ → ^3^H_6_ transition of Tm^3+^ was observed for sample 1Ge-1Ti, where the molar ratio GeO_2_ to TiO_2_ is close 1:1. The energy level diagram for Yb^3+^/Tm^3+^ co-doped titanate–germanate glasses is shown in Figure 4.

In contrast to the Yb^3+^/Er^3+^ system, the energy mismatch between interacting ^2^F_5/2_ (Yb^3+^) and ^3^H_5_ (Tm^3+^) excited levels is much higher [49] and thus, non-resonant Yb^3+^ → Tm^3+^ energy transfer process in titanate–germanate glasses occurs. The excitation energy is transferred nonradiatively by multiphonon relaxation from the ^3^H_5_ level to the lower-lying ^3^H_5_ level generating near-IR emission at 1.8 μm due to ^3^F_4_ → ^3^H_6_ transition of Tm^3+^. Nearly the same mechanism was proposed for Yb^3+^/Ho^3+^ co-doped glass systems, which are also interesting from the optical point of view [50].

**Figure 5:**
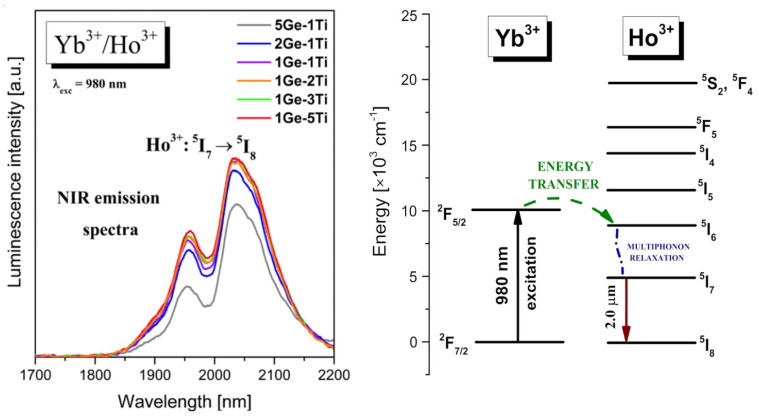
Near-infrared emission spectra and energy level diagram for titanate–germanate glasses co-doped with Yb^3+^/Ho^3+^.

These glass systems present near-IR emission at 2 μm due to the ^5^I_7_ → ^5^I_8_ transition of Ho^3+^ [51,52]. The energy transfer mechanism has been mentioned in the literature by Wang et al. [53]. Upon excitation wavelength at 980 nm, the excited level of Yb^3+^ is well populated and then the excitation energy is transferred from the ^2^F_5/2_ state of Yb^3+^ to the ^5^I_6_ state of Ho^3+^. The energy transfer process Yb^3+^ → Ho^3+^ is non-resonant. In the next step, very fast multiphoton relaxation to the lower-lying ^5^I_7_ level of Ho^3+^ is observed and consequently, we can observe near-IR emission at about 2000 nm associated with the ^5^I_7_ → ^5^I_8_ transition of Ho^3+^ [54,55]. Figure 5 presents near-IR luminescence spectra measured for titanate–germanate glasses co-doped with Yb^3+^/Ho^3+^. The emission bands are more intense for glass samples containing higher concentrations of TiO_2_. All transitions are also indicated in the energy level diagram, which is shown in Figure 5.

**Figure 6:**
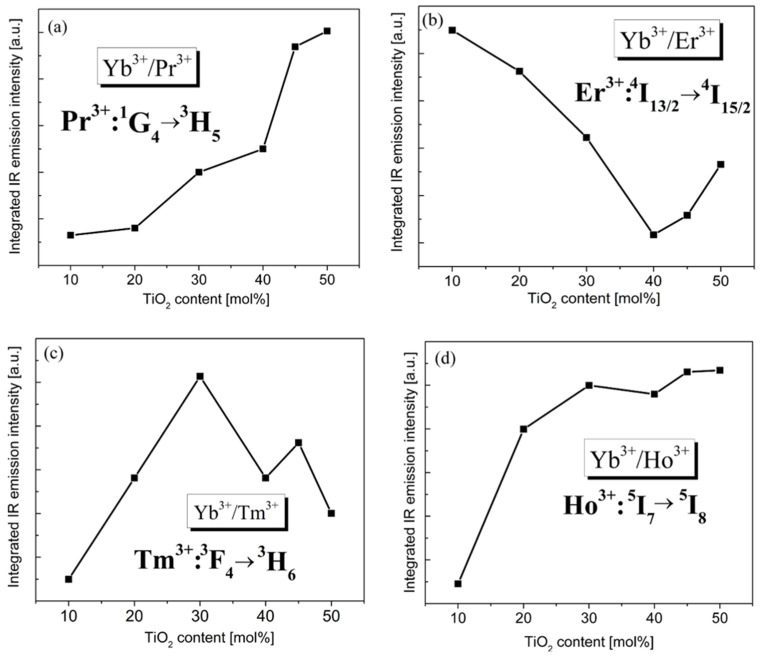
The integrated intensities of emission bands of rare-earth ions Yb^3+^/Pr^3+^ (**a**), Yb^3+^/Er^3+^ (**b**), Yb^3+^/Tm^3+^ (**c**) and Yb^3+^/Ho^3+^ (**d**) vary with TiO_2_.

In order to achieve intense IR emission of rare-earth ions, the heavy doping of the activators such as Pr^3+^, Er^3+^, Ho^3+^, and Tm^3+^ is usually required. Remarkably interesting results were presented in work by Tu et al. [56], where they successfully developed heavily Tm^3+^-doped germanate glasses, promising for glass fibers. On the other hand, the concentrations of activators should be optimal and relatively low in order to reduce luminescence quenching. In our case, luminescence quenching in the studied glass samples is negligibly small because of the low concentrations of acceptors (Pr^3+^, Er^3+^, Ho^3+^, Tm^3+^) and the lack of energy transfer processes between pairs of Pr^3+^-Pr^3+^, Er^3+^-Er^3+^, Tm^3+^-Tm^3+^, and Ho^3+^-Ho^3+^ ions, respectively. The non-radiative transfer processes become dominant for glass samples with higher Ln^3+^ concentrations. These effects are especially stronger for glass systems with diagrams of excited states favoring the presence of cross-relaxation processes. Thus, the probabilities of these non-radiative relaxation processes increase and the luminescence is quenched due to the increasing interaction among the Ln^3+^ ions at higher concentrations. Our spectroscopic investigations indicate that the relative intensities of emission bands of rare-earth ions in germanate glasses are changed drastically with the presence of TiO_2_. Figure 6 shows the integrated intensities of emission bands related to the main ^1^G_4_ → ^3^H_5_ (Pr^3+^),^4^I_13/2_ → ^4^I_15/2_ (Er^3+^),^3^F_4_ → ^3^H_6_ (Tm^3+^) and ^5^I_7_ → ^5^I_8_ (Ho^3+^) near-IR transitions of rare-earth ions in the studied glass samples varying with TiO_2_ content. For pairs Yb^3+^/Pr^3+^ and Yb^3+^/Ho^3+^, the integrated intensities of near-infrared emission bands located at 1.35 and 2 µm increase with increasing TiO_2_ concentration. A completely different situation is observed for pairs of Yb^3+^/Er^3+^ and Yb^3+^/Tm^3+^ ions in titanate–germanate glasses. The integrated intensities of near-infrared emission bands due to the ^4^I_13/2_ → ^4^I_15/2_ transition of Er^3+^ ions are reduced from 5Ge-1Ti to 1Ge-2Ti and then increase with further increasing TiO_2_ content. Contrary to Yb^3+^/Er^3+^, the emission intensities of near-infrared bands related to the ^3^F_4_ → ^3^H_6_ transition of Tm^3+^ ions are enhanced to the 1Ge-1Ti system and then start to decrease with increasing TiO_2_ content in the glass composition; however, the changes in emission intensities with TIO_2_ content are non-linear for pair Yb^3+^/Tm^3+^.

To summarize this part of the research, the authors declare that near-IR emission studies will be devoted in the future to further optimization of the TiO_2_ content of individual systems containing Yb^3+^/Ln^3+^ (Ln = Pr, Er, Tm, Ho). Obtained results for near-IR emission presented here will contribute to the fabrication of titanate–germanate optical fibers.

### 3.3. Luminescence Decays and Energy Transfer Efficiencies

**Figure 7:**
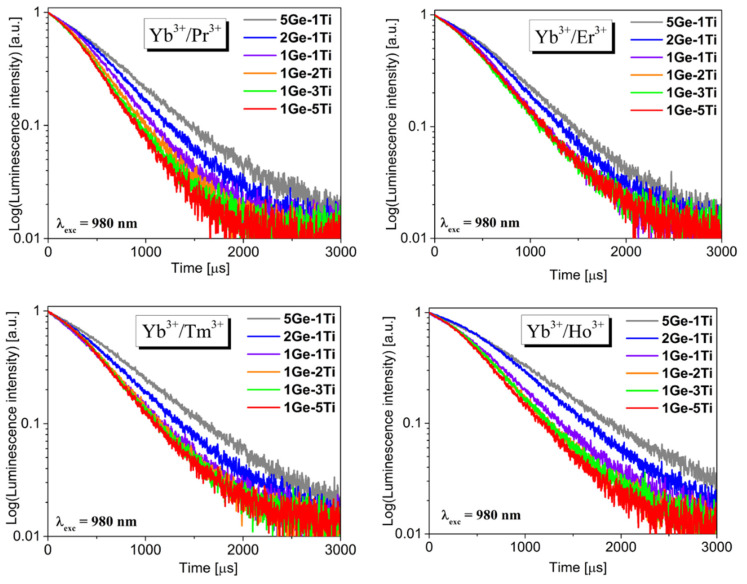
Luminescence decay curves for co-doped titanate–germanate glasses (λ_exc_ = 980 nm).

The systematic studies indicate that luminescence lifetimes for excited states of Yb^3+^ in several low-phonon glass systems are completely different and depend significantly on the glass network-former and network-modifier added to the base composition [57,58]. To determine the efficiency of the energy transfer process between Yb^3+^ and Ln^3+^ ions (Ln = Pr, Er, Tm, Ho), the luminescence decays for titanate–germanate glasses were measured and analyzed. Figure 7 shows decay curves measured for co-doped samples under 980 nm excitation. Based on decays, luminescence lifetimes were determined and compared to Yb^3+^ singly doped glass samples. The results are given in Table 1.

**Figure 8:**
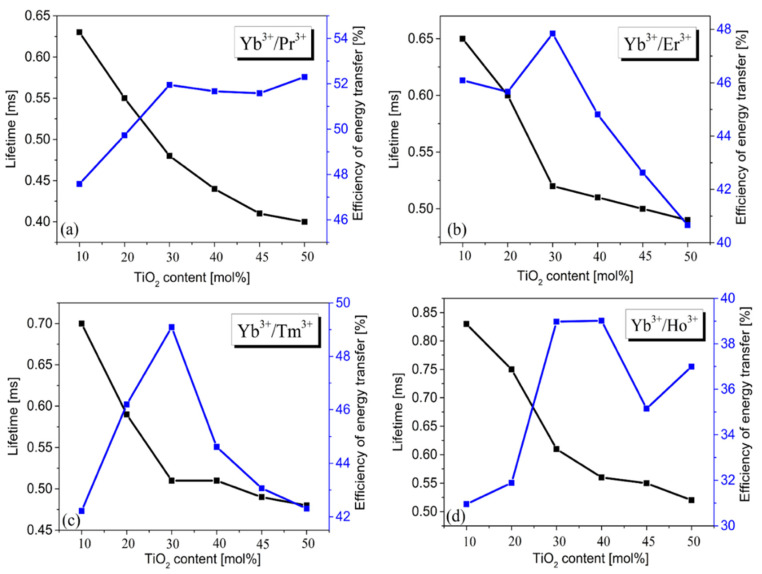
The measured luminescence lifetime and the energy transfer efficiency Yb^3+^ → Ln^3+^ (where Ln = Pr (**a**), Er (**b**), Tm (**c**), Ho (**d**) in the function of TiO_2_ content.

In general, measured lifetimes are reduced from 5Ge-1Ti to 1Ge-5Ti with increasing TiO_2_ concentration in the glass composition. The experimental values of emission lifetimes decrease from 0.63 ms (5Ge-1Ti) to 0.40 ms (1Ge-5Ti) for Yb^3+^/Pr^3+^ co-doped glass systems, 0.65 ms (5Ge-1Ti) to 0.49 ms (1Ge-5Ti) for Yb^3+^/Er^3+^ systems, 0.70 ms (5Ge-1Ti) to 0.48 ms for Yb^3+^/Tm^3+^ systems, and 0.83 ms (5Ge-1Ti) to 0.52 ms (1Ge-5Ti) for Yb^3+^/Ho^3+^ systems, respectively. Luminescence lifetimes measured for Yb^3+^ singly doped glasses and samples co-doped with Yb^3+^/Ln^3+^ were applied to calculate the energy transfer efficiencies [59]. The energy transfer efficiency η_ET_ between Yb^3+^ and lanthanides ions in fabricated glasses was evaluated by calculations with the formula given below:

$$\eta_{\mathrm{ET}}=1\;-\frac{\tau_{\mathrm{Yb}\left(\mathrm{Ln}\right)}}{\tau_{\mathrm{Yb}}}$$

where $\tau_{\mathrm{Yb}\left(\mathrm{Ln}\right)}$ and $\tau_{\mathrm{Yb}}$ are the measured lifetimes for the ^2^F_5/2_ level of Yb^3+^ ions in the presence and absence of acceptor Ln (where Ln = Pr, Er, Tm, Ho), respectively. The results are presented schematically in Figure 8.

Our studies indicate that measured lifetimes decrease with increasing TiO_2_ content, while changes in the energy transfer efficiency seems to be completely different. For all pairs of Yb^3+^/Ln^3+^ (Ln = Pr, Er, Tm, Ho), the energy transfer efficiency is the highest for the 1Ge-1Ti system, but the trend of η_ET_ values varying with TiO_2_ content is not the same. For the pair of Yb^3+^/Pr^3+^, the values of η_ET_ increase to 1Ge-1Ti, whereas they are nearly independent for glasses with higher TiO_2_ content. For pairs Yb^3+^/Er^3+^ and Yb^3+^/Tm^3+^, the energy transfer efficiency increases from 5Ge-1Ti to 1Ge-1Ti and then decreases to 1Ge-5Ti with further increasing TiO_2_ concentration. For the pair of Yb^3+^/Ho^3+^, the values of η_ET_ are the highest for 1Ge-1Ti to 1Ge-2Ti glass systems, respectively; however, the changes of η_ET_ with TiO_2_ content are non-linear. Our calculations indicate that the energy transfer efficiencies are changed from 31% for Yb^3+^/Ho^3+^ glass (5Ge-1Ti) to nearly 53% for Yb^3+^/Pr^3+^ glass (1Ge-5Ti). At this moment, it should also be mentioned that the up-conversion luminescence pathways [60] make an important contribution to the energy transfer processes and their efficiencies in Yb^3+^/Ln^3+^ (Ln = Pr, Er, Ho, Tm) co-doped glasses.

## 4. Conclusions

Multicomponent titanate–germanate glasses co-doped with Yb^3+^/Ln^3+^ (Ln = Pr^3+^, Er^3+^, Tm^3+^, Ho^3+^) were synthesized and then studied their near-IR luminescence properties. The spectroscopic properties of glasses have been examined under the excitation of Yb^3+^ ions by 980 nm. Obtained results were discussed based on the energy level diagrams for sensitizer (Yb^3+^) and acceptors (Pr^3+^, Er^3+^, Tm^3+^, Ho^3+^) and interactions between them. The near-IR luminescence bands corresponding to the ^1^G_4_ → ^3^H_5_ (Pr^3+^), ^4^I_13/2_ → ^4^I_15/2_ (Er^3+^), ^3^F_4_ → ^3^H_6_ (Tm^3+^) and ^5^I_7_ → ^5^I_8_ transitions of lanthanide ions have been examined with TiO_2_ concentration. Our investigations indicate that the intensities of emissions are dependent on titanium dioxide content. The resonant Yb^3+^ → Pr^3+^ and Yb^3+^ → Er^3+^ and non-resonant Yb^3+^ → Tm^3+^ and Yb^3+^ → Ho^3+^ energy transfer process in co-doped titanate–germanate is observed. The analysis of decay profiles allowed for the deeper optical characterization of the energy transfer processes between Yb^3+^ and Ln^3+^ ions (Ln = Pr, Er, Tm, Ho) and for establishing the relation between luminescence lifetimes and the role of titanium dioxide in germanate glasses. Based on decay measurements and values of luminescence lifetimes, the efficiencies of energy transfer were estimated. The values of η_ET_ are changed from 31% for Yb^3+^/Ho^3+^ to nearly 53% for Yb^3+^/Pr^3+^. For all studied pairs Yb^3+^/Ln^3+^, the maximal values of η_ET_ are 53% (Yb^3+^/Pr^3+^), 48% (Yb^3+^/Er^3+^), 49%, (Yb^3+^/Tm^3+^), and 40% (Yb^3+^/Ho^3+^). Further studies revealed that the luminescence lifetimes are reduced with increasing TiO_2_ content, whereas the energy transfer efficiencies are changed completely different, depending on pair Yb^3+^/Ln^3+^ (Ln = Pr^3+^, Er^3+^, Tm^3+^, Ho^3+^) in titanate–germanate glass.

## Figures and Tables

**Table 1:** Measured lifetimes for ^5^F_2_ state of Yb^3+^ in single and co-doped titanate–germanate glasses.

			τ_m_(ms)			
TiO_2_ (mol%)	GeO_2_:TiO_2_	Yb^3+^	Yb^3+^/Pr^3+^	Yb^3+^/Er^3+^	Yb^3+^/Tm^3+^	Yb^3+^/Ho^3+^
10	5:1	1.21	0.63	0.65	0.70	0.83
20	2:1	1.10	0.55	0.59	0.59	0.75
30	1:1	1.00	0.48	0.52	0.51	0.61
40	1:2	1.91	0.44	0.51	0.51	0.56
45	1:3	0.86	0.42	0.50	0.49	0.54
55	1:5	0.84	0.40	0.49	0.48	0.52

## Data Availability

Not applicable.
